# Supplementary material for: Effects of Sodium Nitrate and Coated Methionine on Lactation Performance, Rumen Fermentation Characteristics, Amino Acid Metabolism, and Microbial Communities in Lactating Buffaloes
Source: Microorganisms. 2023 Mar 7;11(3):675. doi: 10.3390/microorganisms11030675 (PMC10057408; doi:10.3390/microorganisms11030675)
Supplement: Supplementary file 1 [file microorganisms-11-00675-s001.zip › microorganisms-2185397-supplementary.pdf]

Table S1. Relative abundance of different bacteria phyla across different treatment groups %.

Taxonomic level	Microbes	CON	SN	MET	SN+MET	SEM	*p* value
Phylum	Bacteroidota	74.63^{a}	71.37^{a}	71.65^{a}	37.76^{b}	3.02	0.004
	Firmicutes	15.91	19.96	18.11	23.64	2.87	0.656
	Proteobacteria	3.69^{b}	2.32^{b}	4.36^{b}	25.92^{a}	1.93	0.004
	Actinobacteriota	0.10^{b}	0.12^{b}	0.17^{b}	8.31^{a}	0.72	0.004
	Cyanobacteria	2.02	2.05	1.56	1.13	0.19	0.133
	Spirochaetota	1.50^{b}	1.25^{b}	2.46^{a}	0.85^{c}	1.17	0.011
	Verrucomicrobiota	1.01	1.30	0.62	0.98	0.29	0.420
Genus	*Prevotella*	60.69^{a}	51.61^{a}	60.67^{a}	24.88^{b}	2.38	0.007
	Acinetobacter	0.09^{b}	0.17^{b}	0.49^{b}	18.46^{a}	1.73	0.001
	Prevotellaceae_UCG-001	3.35^{a}	3.42^{a}	2.78^{a}	1.60^{b}	0.23	0.016
	Rikenellaceae_RC9_gut_group	2.31^{b}	4.01^{a}	1.71^{b}	1.33^{b}	0.35	0.024
	Bacillus	0.03^{b}	0.02^{b}	0.06^{b}	4.36^{a}	0.62	0.004
	norank_f__F082	1.89	3.15	1.11	1.03	0.38	0.062
	Corynebacterium	0.02^{b}	0.02^{b}	0.04^{b}	4.32^{a}	0.57	0.004
	norank_f__norank_o__Gastranaerophilales	2.02	2.04	1.56	1.13	0.32	0.133
	Succiniclasticum	2.06^{a}	1.98^{a}	1.69^{a}	0.71^{b}	0.22	0.048
	norank_f__UCG-011	1.65^{b}	1.68^{b}	2.60^{a}	0.74^{c}	0.38	0.024
	Kurthia	0.04^{b}	0.03^{b}	0.03^{b}	3.37^{a}	0.97	0.017
	Prevotellaceae_UCG-003	1.68^{a}	2.18^{a}	0.95^{b}	0.91^{b}	1.97	0.020
	Pedobacter	0.00^{b}	0.00^{b}	0.00^{b}	3.06^{a}	0.39	0.000
	Treponema	1.35^{a}	0.98^{b}	2.25^{a}	0.75^{b}	0.19	0.018
	Acetobacter	2.68	0.87	1.96	0.28	0.69	0.075
	Christensenellaceae_R	0.81^{a}	1.47^{a}	0.97^{a}	0.39^{b}	0.12	0.017

-7_group						
Butyrivibrio	0.80^{b}	0.65^{b}	1.31^{a}	0.58^{b}	0.12	0.031
NK4A214_group	0.67^{a}	1.44^{a}	0.82^{a}	0.31^{b}	0.26	0.011
norank_f__Bacteroidales_RF16_group	1.01^{a}	1.38^{a}	0.29^{b}	0.40^{b}	0.20	0.009
Lactococcus	0.01^{b}	0.01^{b}	0.01^{b}	1.58^{a}	0.46	0.001
Microbacterium	0.01^{b}	0.01^{b}	0.01^{b}	1.50^{a}	0.13	0.001
Chryseobacterium	0.04^{b}	0.07^{b}	0.33^{b}	1.21^{a}	0.15	0.001
Klebsiella	0.02^{b}	0.04^{b}	0.34^{b}	1.30^{a}	0.36	0.000
Staphylococcus	0.01	0.01^{b}	0.05^{b}	1.40^{a}	0.25	0.001
Enhydrobacter	0.01^{b}	0.01^{b}	0.01^{b}	1.36^{a}	0.09	0.000
Pseudomonas	0.01^{b}	0.01^{b}	0.03^{b}	1.20^{a}	0.09	0.000
norank_f__p-251-o5	0.29	1.17	0.09	0.11	0.06	0.114
Solibacillus	0.01^{b}	0.01^{b}	0.01^{b}	0.98^{a}	0.24	0.001

Values with different superscripts in the same row differ significantly. CON was fed on a total mixed ratio (TMR), SN was fed on TMR supplemented with 70 g/d sodium nitrate, MET was fed on TMR supplemented with 15 g/d L-methionine, SN+MET was fed on TMR supplemented with sodium nitrate group 70 g/d + L-methionine 15 g/d.
